# Editorial: Artificial intelligence in infectious diseases: pathogenesis and therapy

**DOI:** 10.3389/fmed.2024.1414056

**Published:** 2024-05-14

**Authors:** Jason C. Hsu, Christine Y. Lu, Min-Huei Hsu

**Affiliations:** ^1^International Ph.D. Program in Biotech and Healthcare Management, College of Management, Taipei Medical University, Taipei, Taiwan; ^2^Clinical Big Data Research Center, Taipei Medical University Hospital, Taipei Medical University, Taipei, Taiwan; ^3^Clinical Data Center, Office of Data Science, Taipei Medical University, Taipei, Taiwan; ^4^Research Center of Data Science on Healthcare Industry, College of Management, Taipei Medical University, Taipei, Taiwan; ^5^Kolling Institute, Faculty of Medicine and Health, The University of Sydney and the Northern Sydney Local Health District, Sydney, NSW, Australia; ^6^School of Pharmacy, Faculty of Medicine and Health, The University of Sydney, Sydney, NSW, Australia; ^7^Department of Population Medicine, Harvard Medical School, Boston, MA, United States; ^8^Graduate Institute of Data Science, College of Management, Taipei Medical University, Taipei, Taiwan

**Keywords:** artificial intelligence, infectious diseases, COVID-19, Machine Learning, prediction model

In recent years, the use of artificial intelligence (AI) in the medical field has shown promising potential in revolutionizing the understanding, diagnosis, and treatment of infectious diseases. This editorial delves into how AI, through various studies, is reshaping the landscape of pathogenesis and therapy in the realm of infectious diseases, notably COVID-19, Pulmonary Tuberculosis, and Hansen's Disease, commonly known as leprosy.

The systematic review by Shakibfar, Nyberg et al. is a cornerstone in understanding AI's application in predicting COVID-19 outcomes. The study highlights the reliance on Random Forest models, which, despite their moderate accuracy, face challenges regarding bias risk and limited external validation. This underscores the critical need for thorough validation processes to ensure AI's reliability in healthcare predictions. Another study by Shakibfar, Zhao et al. extends AI's potential by developing a Disease Risk Score for COVID-19 hospitalization and mortality using health registry data, demonstrating its utility in identifying high-risk individuals, albeit with certain limitations in generalizability and external validation.

Expanding the horizon, Banoei et al. leverage Machine Learning (ML) models to predict mortality among hospitalized COVID-19 patients. By analyzing complex relationships between various risk factors, the study identifies significant predictors of mortality, such as low oxygen saturation and chronic kidney disease, thus providing a foundation for understanding interactions between risk factors and prioritizing treatment. Similarly, Hien et al. utilize real-world clinical data to develop predictive models for severe COVID-19 outcomes, showcasing the potential of ML in aiding early identification and management of high-risk patients. Subsequent research further advances the exploration of risk factors and predictive modeling for other infectious diseases.

Building on the analysis of risk factors and prediction model, the study by Gao et al. transitions us to Pulmonary Tuberculosis. They introduce TBINet, a deep learning model utilizing CT images to distinguish Pulmonary Tuberculosis infectivity. This innovative approach, validated by superior AUC values and Grad-CAM technology, presents a rapid and reliable method for diagnosis and control of Pulmonary Tuberculosis, demonstrating AI's usefulness in medical imaging analysis.

Lastly, the article by Deps et al. brings to light AI's role in managing Hansen's Disease, addressing challenges such as late diagnoses and underdiagnosis. AI's capabilities in rapid case detection, personalized treatment, and stigma combat through mental health counseling highlight the transformative impact AI can have in therapeutic approaches, significantly reducing the burden of Hansen's disease.

In conclusion, the integration of AI into the clinical management of infectious diseases holds immense promise in reshaping our approach to diagnosis, treatment, and understanding of pathogenesis. The studies reviewed herein not only underscore AI's potential in enhancing academic knowledge and clinical practices but also hint at the need for policy adjustments to accommodate and leverage AI's capabilities fully. As we venture further into this AI-driven era, the convergence of technology and healthcare beckons a future where infectious diseases are managed more efficiently, paving the way for innovative therapeutic strategies and improved patient outcomes.

## Author contributions

JH: Conceptualization, Project administration, Writing – original draft, Writing – review & editing. CL: Writing – original draft, Writing – review & editing. M-HH: Writing – review & editing.

